# Arsenic trioxide rewires mantle cell lymphoma response to bortezomib

**DOI:** 10.1002/cam4.511

**Published:** 2015-08-26

**Authors:** Ling-Ling Zhao, Yuan-Fang Liu, Li-Jun Peng, Ai-Mei Fei, Wen Cui, Sheng-Chao Miao, Olivier Hermine, Remy Gressin, Saadi Khochbin, Sai-Juan Chen, Jin Wang, Jian-Qing Mi

**Affiliations:** 1 State Key Laboratory for Medical Genomics and Department of Hematology, Shanghai Institute of Hematology, Collaborative Innovation Center of Systems Biomedicine, Pôle Sino-Français des Sciences du Vivant et Genomique, Rui Jin Hospital, Shanghai Jiao Tong University School of MedicineShanghai, China; 2 Department of Clinical Laboratory, Shanghai Xuhui Central HospitalShanghai, China; 3 Service d’Hématologie Adultes, Hôpital Necker-Enfants Malades, Assistance Publique-Hôpitaux de Paris, Université Paris DescartesParis, France; 4 Département d’Onco-Hématologie, Hôpital A Michallon, CHU de GrenobleGrenoble, France; 5 INSERM U823, Institut Albert Bonniot, Faculté de Médecine, Université Grenoble AlpesLa Tronche, France

**Keywords:** Arsenic trioxide, bortezomib, mantle cell lymphoma, Mcl-1, Noxa

## Abstract

Although most of the mantle cell lymphoma (MCL) patients initially responded well to bortezomib (BTZ), the dose-dependent toxicities have greatly limited the application of BTZ to MCL. To investigate the efficacy and mechanism of arsenic trioxide (ATO) with BTZ in inducing apoptosis of MCL cells, two MCL cell lines, along with primary cells from MCL patients (*n* = 4), were used. Additionally, the NOD-SCID mice xenograft model of Jeko-1 cells was established to study the anti-MCL mechanisms in an in vivo setting. ATO treatment highly improved BTZ capacity to inhibit proliferation and induce apoptosis of MCL cells. Furthermore, the interaction of Noxa and Mcl-1 leads Bak to release from Mcl-1 or from Bcl-xl, which could further activate Bak and Bax and then induce cell apoptosis. We also found that when lower doses of BTZ were used in combination with ATO, more effective proapoptotic effects in both the cell lines and the primary cells were obtained compared to the effects of BTZ used alone at higher doses. Simultaneously, the combination of these two drugs delayed the tumor growth in mice more effectively than BTZ alone. The cooperative anti-MCL effects of this combination therapy both in vitro and in vivo strongly provided a new strategy to the clinical treatment of MCL.

## Introduction

Mantle cell lymphoma (MCL) is a subtype of non-Hodgkin’s lymphoma originated from germinal center B cells, comprising 5–10% of all lymphomas 1. It is mainly characterized by the cyclin D1 overexpression induced by t (11;14) (q13;q32) translocation. Although MCL responses well to traditional chemotherapies and a higher response rate can be achieved, relapse within a few years after remission is still a common outcome 2–5. Therefore, the prognosis of MCL is generally quite poor, with a median survival of only 5–7 years 6,7.

The overall response rates of relapsed and refractory MCL patients treated with proteasome inhibitor bortezomib (BTZ) monotherapy are quite high (29–50%) 8–11, however, severe peripheral neuropathies occur during BTZ treatment. Patients usually present peripheral neuropathic pain, feeling of numbness in distal extremities, even being unable to walk or becoming paralyzed 12–15. Up to now, the most successful method of preventing neurotoxicity is still dose reduction or withdrawal of BTZ. Unfortunately, the response rates and duration of response also decrease along with the dose reduction of BTZ, which challenges treatment of MCL by BTZ 16–19.

Studies have shown that antitumor effect of BTZ on MCL cells is primarily exerted by increasing the proapoptotic protein Noxa; however, the accumulation of antiapoptotic protein Mcl-1 induced by inhibition of intracellular proteasome activity via BTZ weakens the antitumor effect of the drug 20–23. Therefore, combined therapy with another reagent, which decreases the level of antiapoptotic protein Mcl-1, while maintaining the high level of proapoptotic protein Noxa, may be the best strategy of BTZ treatment to MCL.

Our center has been focusing on revealing the mechanism of arsenic trioxide (ATO) therapy on acute promyelocytic leukemia (APL) for two decades, with the discovery of ATO being able to induce apoptosis of APL cells through multiple molecular mechanisms 24,25. Recently, we found for the first time that ATO can downregulate the level of Mcl-1 in MCL cells 26, which suggested that combined use of ATO and BTZ may not only increase the proapoptotic protein Noxa but also reduce the antiapoptotic protein Mcl-1, and thereby play a synergistic effect on induction of apoptosis in MCL cells.

In 2012, Jung et al. reported that ATO and BTZ could exert synergistic effects on MCL, which confirmed our hypothesis. They showed that ATO could significantly enhance the antiproliferative effects of BTZ on MCL. As to the mechanism, they confirmed that ATO treatment also reduced cyclin D1 expression and also suppressed the NF-*κ*B activation in MCL cell lines 27. We attempted to clarify the mechanisms of regulation of Noxa/Mcl-1 by ATO combined with BTZ, so as to complement their work mentioned above. It is of note that reports on the in vivo effects of ATO combined with BTZ are still lacking.

This research program investigated the synergistic role of BTZ with ATO to MCL cells both in vitro and in vivo, providing theoretical and practical basis for the improvement of MCL treatment.

## Materials and Methods

### Cell lines and primary cells

The MCL cell lines Jeko-1 (ATCC, Manassas, VA) and Granta-519 (DSMZ, Braunschweig, Germany) were cultured in RPMI 1640 culture medium (Gibco, Paisley, Scotland, United Kingdom), supplemented with 10% heat-inactivated fetal bovine serum (FBS) (Biochrom AG, Berlin, Germany), 2 mmol/L l-glutamine, 100 units/mL of penicillin G, and 10 mg/mL of streptomycin (Gibco). Following informed consent, bone marrow samples from four previously untreated MCL patients with leukemic disease were obtained and all cases had a translocation t (11;14) (q13;q32). Bone marrow mononuclear cells (BMMCs) from MCL patients and peripheral blood mononuclear cells (PBMCs) from five adult healthy donors were isolated by gradient centrifugation using Lymphoprep (Axis-Shield, Oslo, Norway). Purified primary cells were used immediately. All MCL cell lines and primary cells were cultured at 37°C in a humidified atmosphere containing 5% carbon dioxide. Cells were incubated for 12–48 h single or combined of BTZ (Millennium Pharmaceuticals, Cambridge, MA) and ATO (the Pharmacy of Chinese Traditional Medicine in the First Hospital affiliated to Harbin Medical University). When pan-caspase inhibitor z-VAD-FMK (Beyotime, Haimen, China) was used, cells were preincubated for 1 h prior to the addition of BTZ and ATO. An approval was obtained from the Institutional Review Board of Shanghai Jiao Tong University School of Medicine.

### Cell viability assay

Cell viability was assessed using the Cell Counting Kit-8 (Dojindo, Tokyo, Japan). Cells were seeded into a 96-well plate at a density of 2 × 10^4^ cells per 100 *μ*L medium per well and treated with BTZ, ATO, or combination at various concentrations and normal saline (NS) as vehicle for 12, 24, and 48 h. CCK8 (10 *μ*L) solution was then added to each well and the plates were incubated at 37°C for 2–4 h. Optical density (OD) was measured by a microplate reader at 450 nm. The cell inhibition rate was calculated according to the following equation:

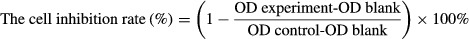
All experiments were done in triplicate and repeated three independent times.

### Flow cytometry detection of apoptosis features

Phosphatidylserine exposure was quantified by annexin V–FITC apoptosis detection kit I (BD Biosciences, San Diego, CA) according to the manufacturer’s instruction. For the analysis of apoptosis of BMMCs from MCL patients, 1, 2, 3, annexin V–PE apoptosis detection kit I (BD) was used. Before staining with annexin V–PE/7AAD, cells were labeled with CD45-PE-cy7/CD45-PO, CD5-FITC, and CD19-PB (Beckman Coulter, Miami, FL) and cells were then washed and resuspended in annexin-binding buffer. Changes in mitochondrial transmembrane potential (Δ*ψ*m) were evaluated by staining cells with 20 nmol/L DiOC6 (Molecular Probes, Eugene, OR) for 20 min at room temperature in the dark, cells were then washed and resuspended in 1 × phosphate buffered saline (PBS). A total of 10,000 cells per sample were acquired in a FACScan flow cytometer (BD). Experiments were performed in triplicate and repeated three independent times.

### Flow cytometric analysis for Bak and Bax activation

Cells were fixed with 4% paraformaldehyde for 10 min at 37°C and permeabilized with 90% methanol for 30 min on ice, then washed and resuspended in incubation buffer (0.5% BSA). After fixation and permeabilization, cells were incubated with anti-Bax (clone 6A7; BD), anti-Bak (clone Ab-1; Millipore, Billerica, MA, or IgG1 isotype control for 30 min at room temperature, followed by goat anti-mouse FITC (Dako, Glostrup, Denmark), and were analyzed in a FACScan flow cytometer. Experiments were performed in triplicate and repeated three independent times.

### Western blot and antibodies

Cells were lysed by RIPA buffer (50 mmol/L Tris [pH 7.4], 150 mmol/L NaCl, 0.1% sodium dodecyl sulfate [SDS], 1% NP-40, 0.5% sodium deoxycholate, sodium orthovanadate, sodium fluoride, ethylenediaminetetraacetic acid (EDTA), leupeptin) containing a mixture of protease inhibitors phenylmethanesulfonyl fluoride (PMSF) and cocktail. Protein extracts were loaded onto a 10–15% SDS-polyacrylamide gel electrophoresis (PAGE) gel and electrotransfer or electroblotting onto Polyvinylidene Fluoride (PVDF) (Millipore) was performed. The blots were incubated with antibodies against cleaved caspase-3, caspase-8, caspase-9, cleaved poly (ADP-ribose) polymerase (PARP) (Cell Signaling), and Mcl-1, Bcl-2 (Santa Cruz, CA), Bcl-xl, Puma, Bim, Bid, Bak (Cell Signaling, Beverly, MA), Noxa (Alexis Biochemical, Lausen, Switzerland), Bax (BD), followed by incubation with horseradish peroxidase-linked secondary antibody (Cell Signaling). Membranes were developed with enhanced chemiluminiscence substrate (Millipore), and the signal was revealed on LAS4000 Fuji film, Tokyo, Japan device. Equal protein loading was confirmed with *β*-actin (Sigma, St Louis, MO). Relative protein quantification was done with Quantity One software (Bio-Rad, Hercules, CA). All experiments were repeated three independent times.

### Immunoprecipitation

In order to analyze the Mcl-1/Noxa interaction by Mcl-1 immunoprecipitation, NP-40 buffer was used (0.5% NP-40 [vol/vol], 50 mmol/L 4-(2-Hydroxyethyl)-1-piperazineethanesulfonic acid (HEPES) [pH 7.7], 150 mmol/L NaCl, 0.1 mmol/L EDTA, 1 *μ*g/mL cocktail, and 1 mmol/L PMSF) 20. Protein extracts were incubated overnight at 4°C with 4 *μ*g anti–Mcl-1 antibody (Santa Cruz), Protein A+G beads (Beyotime) were then added for three more hours. Supernatant (nonimmunoprecipitated fraction) was recovered by centrifugation, and Protein A+G beads (immunoprecipitated fraction) were washed five times with NP-40 buffer. Reducing 5× sample buffer (250 mmol/L Tris-HCl pH 6.8; 10% SDS; 0.5% bromophenol blue; 50% glycerol; 5% *β*-mercaptoethanol) was added to both fractions, boiled, and analyzed in 15% polyacrylamide gels followed by western blot. Membranes were probed with monoclonal anti-Noxa (Alexis Biochemicals) and monoclonal anti-Mcl-1 (clone 22; BD) antibodies.

For Mcl-1 and Bcl-xl immunoprecipitation to analyze Mcl-1/Bak and Bcl-xl/Bak interactions, respectively, Pierce Crosslink Immunoprecipitation Kit (Thermo, Rockford, IL) was used according to the manufacturer’s instruction with anti–Mcl-1 (Santa Cruz) and anti–Bcl-xl (Cell Signaling) antibodies. Membranes were probed with polyclonal anti-Bak (NT; Upstate), monoclonal anti–Mcl-1 (clone 22; BD), and anti–Bcl-xl (Cell Signaling) antibodies. All experiments were repeated three independent times.

### In vivo xenograft model

Female NOD–SCID mice (4- to 6-week-old) were housed and monitored in specific pathogen-free facilities at the Department of Laboratory Animal Science, Shanghai Jiao Tong University School of Medicine. All experimental procedures and protocols were approved by Institutional Review Board of Shanghai Jiao Tong University School of Medicine. SCID mice were subcutaneously inoculated in the right flank with 8 × 10^6^ Jeko-1 cells suspended in 100 *μ*L 1× PBS. Tumor burdens were assessed using the two largest perpendicular axes measured with standard calipers, and tumor volume was calculated using the formula: 1/2 (width)^2^ × (length). When palpable tumors (≥5 mm in diameter) developed, mice were randomly separated into four treatment groups of 10 mice each and treated with intraperitoneal injections of either BTZ alone (1 mg/kg on days 1, 4, 11) or ATO alone (2 mg/kg on days 1–5, 8–12, 18–22), or BTZ in combination with ATO, or vehicle (0.9% NaCl). Tumor size and body weight were measured every 2 days and mice were photographed every 2–4 days for the duration of the experiment. Animals were euthanized when the diameter of their one-dimensional tumors reached 15 mm.

### Statistical analysis and drug combination assay

All assays were performed in triplicate, and data are expressed as mean ± SEM. Statistical analyses were performed using software GraphPad Prism, GraphPad, San Diego, CA and SPSS, IBM Corporation, Armonk, NY. Statistical significance of differences between groups was evaluated by Student’s *t*-test or analysis of variance (ANOVA) test. *P *<* *0.05 was considered statistically significant.

Synergy calculations were performed using the effect multiplication principle where expected survival = SA × SB (SA: surviving cell fraction of treatment A, SB: survival cell fraction of treatment B). Synergy occurs when actual survival is less than the expected survival when the two treatments are combined 28.

## Results

### BTZ and ATO cooperatively induce growth arrest of MCL cell lines

Although the inhibitory effect of BTZ on MCL cell proliferation has been well established 21,22,29, in this study, to be stringent, we still confirmed the effect of BTZ on two strains of cell lines, Jeko-1 and Granta-519 (Fig. S1A). BTZ exhibited similar effect on the two cell lines, and the inhibitory effect were proportional to dosage. The dose of BTZ we used were 0, 2.5, 5, 7.5, 10, 15, 20 nmol/L; moreover, similar results were obtained at different time points (12, 24, and 48 h) (Fig. S1A).

Our previous work 26 reported that ATO could inhibit the proliferation of MCL cell lines in a dose-dependent manner. The proliferation of Jeko-1 and Granta-519 cell lines was efficiently inhibited with a concentration of 0, 0.25, 0.5, 1, 2, and 5 *μ*mol/L of ATO at 12, 24, and 48 h (Fig. S1B). The inhibition of cell proliferation became more obvious with dose escalation.

**Figure 1:**
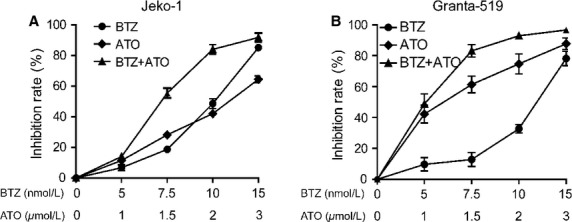
ATO combined with BTZ inhibited the proliferation of MCL cells. MCL cell lines, Jeko-1 (A), Granta-519 (B), were treated by BTZ/ATO in fixed ratio (5/1, 7.5/1.5, 10/2, 15/3 [nmol/L]/[*μ*mol/L]). After 12 h, cell viability was determined by CCK8. ATO, arsenic trioxide; BTZ, bortezomib; MCL, mantle cell lymphoma.

In the experiments of combination therapy, we chose appropriate concentrations of BTZ and ATO and adjusted the ratio of concentrations to ensure that a certain gradient of inhibition effects on cell proliferations could be found when each single drug or the combination therapy were used. We found that when BTZ was given at the effective concentrations (such as 7.5 and 10 nmol/L), the combination with ATO could exert significant synergistic effect (Fig.1).

### BTZ and ATO cooperatively induce apoptosis of MCL cell lines in a caspase-dependent pathway

**Figure 2:**
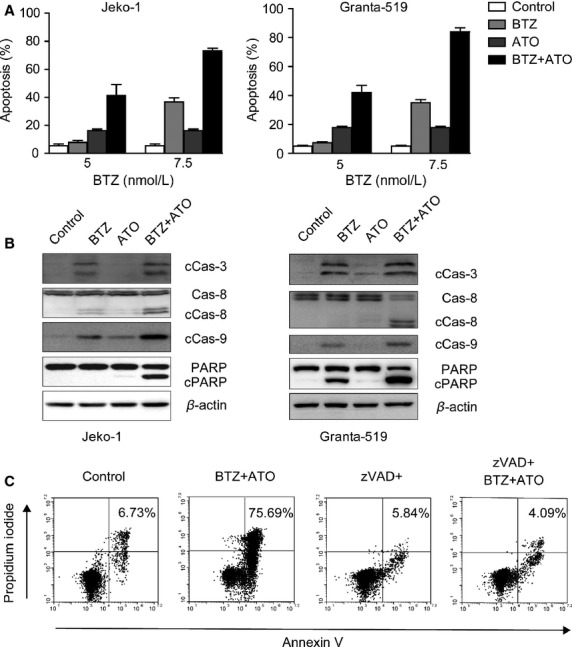
ATO combined with different concentrations of BTZ induced apoptosis of MCL cells. (A) The apoptotic status of MCL cells, 24 h after the Jeko-1 and Granta-519 being treated by 1 *μ*mol/L ATO, 5 or 7.5 nmol/L BTZ, or combination of the two drugs. (B) The expression of apoptosis-related proteins in Jeko-1 and Granta-519 cells detected by western blot, 12 h after treatment of ATO alone, BTZ alone, or combination of the two drugs. (C) The apoptotic status of Jeko-1 cells, after incubation with the caspase inhibitor, zVAD-FMK, for 1 h, followed by treatment of BTZ and ATO for 24 h. ATO, arsenic trioxide; BTZ, bortezomib; MCL, mantle cell lymphoma.

When we treated the three MCL cell lines with combination of BTZ (5 and 7.5 nmol/L) and ATO (1 *μ*mol/L) for 24 h, we observed an obvious cooperative effect (Fig.2A) on each cell line. BTZ used at a concentration of 5 nmol/L along with ATO (1 *μ*mol/L), induced MCL cell apoptosis to the same extent as a treatment by 7.5 nmol/L BTZ alone (*P* > 0.05).

To elucidate the mechanism underlying cell apoptosis after treatment with BTZ, ATO, or combined use of these two drugs, we used 7.5 nmol/L BTZ, 1 *μ*mol/L ATO to treat MCL cells for 24 h, and then analyzed the activation of caspase family proteins and the cleavage of their downstream PARP protein by western blot. After treating with BTZ, ATO alone, or in combination, the apoptosis-related proteins caspase-3, caspase-8, and caspase-9 were activated and PARP was cleaved (Fig.2B).

After 24-h treatment by 7.5 nmol/L BTZ and 1 *μ*mol/L ATO, 75.69% of the Jeko-1 cells underwent apoptosis, while only 6.73% apoptotic cells were detected in the control nontreated cell group. Under the same conditions, pretreatment of cells with pan-caspase inhibitor z-VAD-FMK (100 *μ*mol/L) severely interfered with apoptosis induced by BTZ and ATO (4.09%), which was comparable to the nontreated control cell group (5.84%) (Fig.2C). We have the similar results in the Granta-519 cells.

### BTZ combined with ATO induced loss of mitochondrial transmembrane potential (Δ*ψ*m) and activation of Bak and Bax

**Figure 3:**
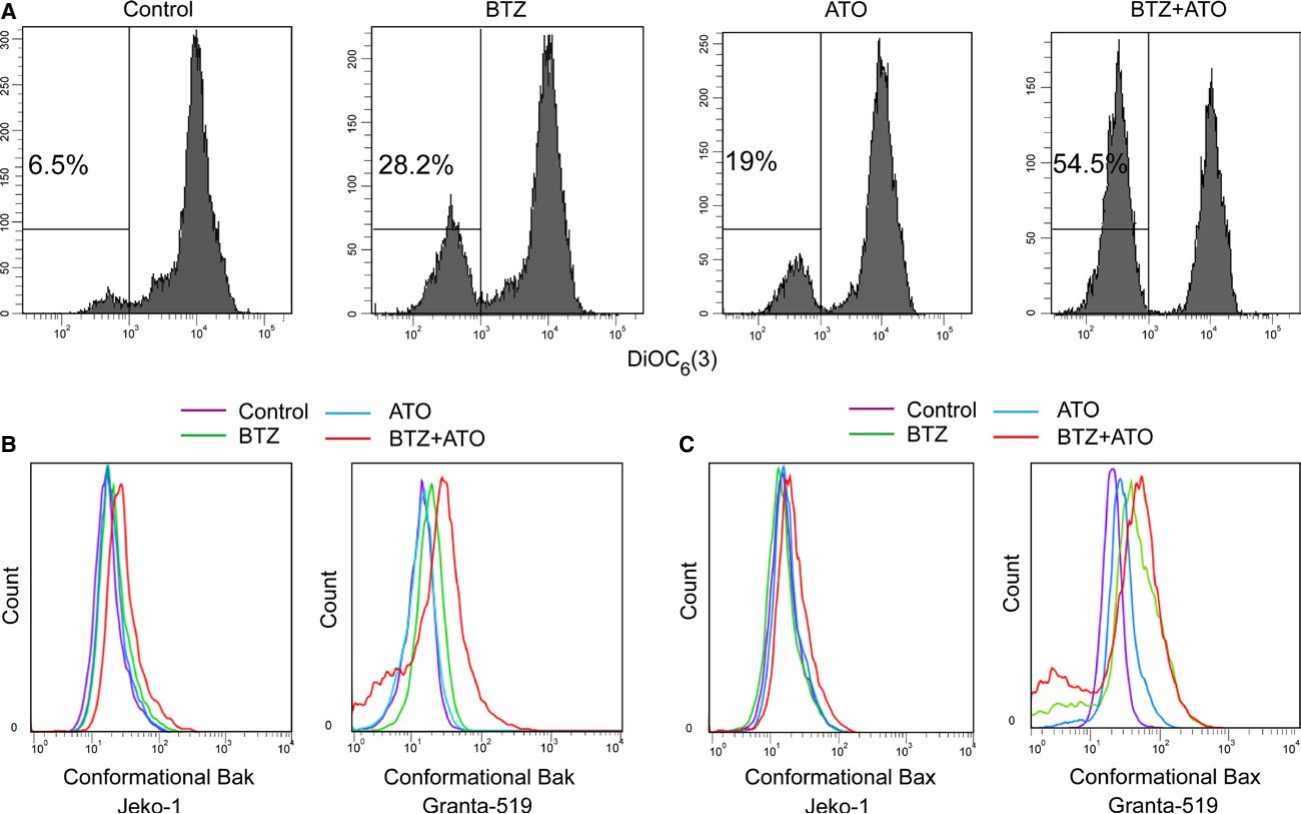
ATO combined with BTZ activated the intrinsic apoptotic pathway of MCL cells. The loss of Δ*ψ*m (A), Bak conformational changes (B), Bax conformational changes (C) were detected after 24-h treatment of ATO, BTZ, and combination of the two drugs. ATO, arsenic trioxide; BTZ, bortezomib; MCL, mantle cell lymphoma.

After incubation with 7.5 nmol/L BTZ or 1 *μ*mol/L ATO or combination of 7.5 nmol/L BTZ and 1 *μ*mol/L ATO, respectively, for 24 h, 28.2% of the Jeko-1 cells lost Δ*ψ*m after incubation with 7.5 nmol/L BTZ alone, 19% of the cells with 1 *μ*mol/L ATO, and 54.5% with combination of these two drugs (6.5% in control) (Fig.3A) and the similar results were detected in the Granta-519 cells. The proportion of cells losing Δ*ψ*m was significantly higher in combined treatment group than that in single drug groups. This illustrated that the cooperative effect of ATO and BTZ played a very important role in mitochondrial pathway of apoptosis in MCL cell lines.

To analyze the activation of Bak and Bax, we used 7.5 nmol/L BTZ, 1 *μ*mol/L ATO, or the two drugs in combination to treat MCL cells for 24 and 48 h, then checked the conformational changes of Bak (Fig.3B) and Bax (Fig.3C), and found that both Bak and Bax did undergo a conformational change in each treatment group, with more profound effects when BTZ and ATO were used together which was particularly evident at 48 h (Fig. S2A and B).

### BTZ and ATO synergistically induce apoptosis by regulating the interaction between Mcl-1 and Noxa

**Figure 4:**
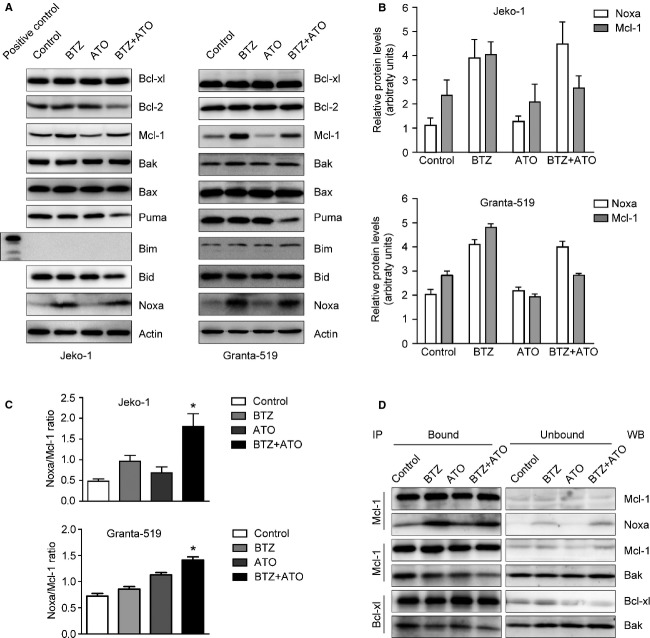
The effect of ATO combined with BTZ on the Bcl-2 family proteins and the release of Bak. Jeko-1 and Granta-519 cells were treated by ATO, BTZ, or combination of the two drugs for 24 h, after that, (A) the expression of Bcl-2 family proteins was detected. One representative experiment is shown from three independent experiments. (B) The protein levels of Noxa and Mcl-1 were quantified by Quantity One software (Bio-Rad). (C) The ratio between expression levels of Noxa and Mcl-1 was showed. *P <* *0.05. (D) The expression levels of Mcl-1, Noxa, Bak, and Bcl-XL proteins in the immunoprecipitated (bound) and nonimmunoprecipitated (unbound) fractions were detected. ATO, arsenic trioxide; BTZ, bortezomib.

The balance between proapoptotic and antiapoptotic Bcl-2 family proteins plays an important role in cell apoptosis. We detected the expression of Bcl-2 family proteins of Jeko-1 cells after treatment of 7.5 nmol/L BTZ alone, 1 *μ*mol/L ATO alone, or the combination of two drugs for 24 h (Fig.4A). The expression of antiapoptotic protein Bcl-xl did not alter significantly after treatment; no apparent change of the protein level of Bcl-2 was found in the BTZ group and ATO group compared with the control cells in two MCL cell lines, whereas in the combination group, the level of Bcl-2 decreased in the Jeko-1 cells and not changed in the Granta-519 cells. Another antiapoptotic protein Mcl-1 increased when treated by BTZ alone, while, on the contrary, Mcl-1 was reduced in the ATO group. Interestingly, ATO treatment could to some extent neutralize the BTZ-induced Mcl-1 accumulation when the two drugs were used in combination (Fig.4A).

Although the proapoptotic protein Bak and Bax underwent conformational changes after treatment (Fig.3B and C; Fig. S2), the total level of proteins did not vary significantly (Fig.4A). The BH3-only protein puma decreased slightly; the level of Bim did not change apparently in the Granta-519 cells, and this expression was not detected in the Jeko-1 cells, since Bim is not expressed 30,31. The combination of BTZ and ATO reduced slightly the protein level of BH3-only protein Bid in the Jeko-1 cells but not in the Granta-519 cells. It is noteworthy mentioning that the BH3-only protein Noxa, which promotes apoptosis by binding to Mcl-1, increased significantly after treatment with BTZ alone or with the combination of the two drugs in both Jeko-1 cells and Granta-519 cells (Fig.4A).

We detected the relative expression levels of Mcl-1 and Noxa of MCL cells in the control group, BTZ monotherapy group, ATO monotherapy group, and two-drug combination group (Fig.4B). On this basis we obtained the ratio between expression levels of Noxa and Mcl-1. This ratio in the two MCL cells was the highest in the two-drug combination group compared with that in single drug or control group (*P* < 0.05, Fig.4C).

In general, Bak released from Mcl-1 and Bcl-xl is determinant for the onset of mitochondrial apoptotic pathway, and the BH3-only proteins, such as Noxa, is responsible for this release 32,33. In addition, the downregulation of Mcl-1 can activate Bak, for example, in the presence of ATO 34.

After incubation with 7.5 nmol/L BTZ, 1 *μ*mol/L ATO, or a combination of these two drugs for 24 h, the Mcl-1 protein in Jeko-1 cells were immunoprecipitated by anti-Mcl-1 mAbs to analyze the Mcl-1/Noxa and Mcl-1/Bak interactions (Fig.4C). Noxa/Mcl-1 complex was very low in the untreated control group, while Bak/Mcl-1 complex was found in a significant amount. In the BTZ group, the binding of Noxa to Mcl-1 significantly increased, accompanied by a decrease in Bak binding to Mcl-1. In the ATO group, no increase was observed in the level of Noxa binding to Mcl-1 compared with control, while ATO treatment resulted in a significant reduction of Bak binding to Mcl-1. In the two-drug combination group, the level of Noxa binding to Mcl-1 also increased significantly, but Bak binding to Mcl-1 was the lowest among the four groups.

At the same time, we analyzed the interaction of Bcl-xl/Bak by immunoprecipitation using anti-Bcl-xl mAb. Bak binding to Bcl-xl decreased significantly after incubation with BTZ, ATO, or BTZ combined with ATO, with the lowest amount of coimmunoprecipitated Bak in the combination group (Fig.4C).

### ATO induces apoptosis cooperatively with BTZ in primary cells of MCL patients

**Figure 5:**
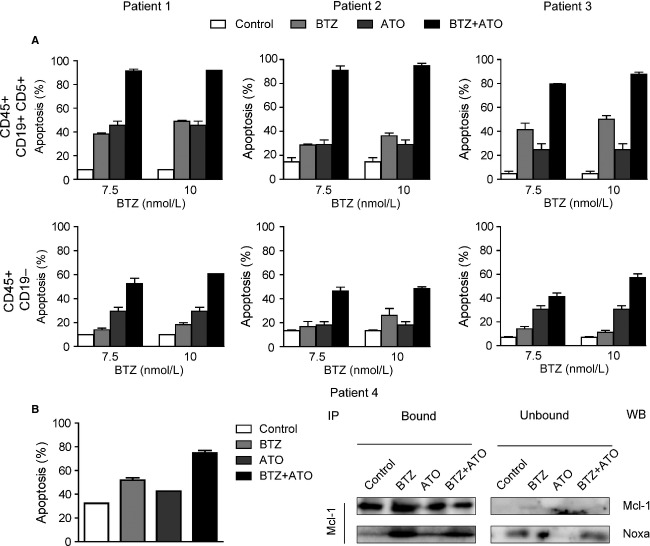
ATO combined with BTZ induced apoptosis in primary cells of MCL patients. (A) The apoptotic status of CD45+CD19+CD5+ and CD45+CD19− cells from BMMCs of patient 1, 2, and 3 were determined after 18-h treatment of 2 *μ*mol/L ATO, 7.5 or 10 nmol/L BTZ, and combination of the two drugs. (B) The apoptotic status of BMMCs of patient 4 was determined after 18-h treatment of 2 *μ*mol/L ATO, 7.5 nmol/L BTZ and combination of them. The protein levels of Mcl-1 and Noxa in immunoprecipitated (bound) and nonimmunoprecipitated (unbound) fractions were detected. ATO, arsenic trioxide; BTZ, bortezomib; MCL, mantle cell lymphoma; BMMCs, bone marrow mononuclear cells.

The effects of BTZ and ATO were further verified in the primary cells from four patients with MCL. In patient 1, 2, and 3, the BMMCs were incubated with different concentrations (7.5, 10 nmol/L) of BTZ, 2 *μ*mol/L ATO, or combination of these two drugs. Eighteen hours later, significant cooperative effects of these two drugs were demonstrated in the tumor cells (CD45+CD19+CD5+ cells). Of note, the cooperative effect of 2 *μ*mol/L ATO and 7.5 nmol/L BTZ on apoptotic induction of MCL cells was significantly higher than 10 nmol/L BTZ monotherapy (*P* < 0.05); while in the nontumor cells (CD45+CD19− cells), the apoptotic induction effect of two-drug combination therapy was weaker (Fig.5A). We also tested the PBMCs of five healthy volunteers. Their apoptosis was considerably lower than that of tumor cells (Fig. S3).

The tumor cells of the fourth patient reached 93.1% of the bone marrow cells, so we treated his BMMCs directly. Apart from confirming the same results as those of the above three patients, we also conducted Mcl-1 immunoprecipitation to examine the interaction between the cellular antiapoptotic protein Mcl-1 and the proapoptotic protein Noxa before and after treatment (Fig.5B). The result was consistent with that in the Jeko-1 cells, the binding of Noxa and Mcl-1 increased after treatment of BTZ alone or both drugs. Unfortunately, Bak level was undetectable due to the amount limitation of the samples.

### The cooperative in vivo effects of ATO and BTZ against MCL

**Figure 6:**
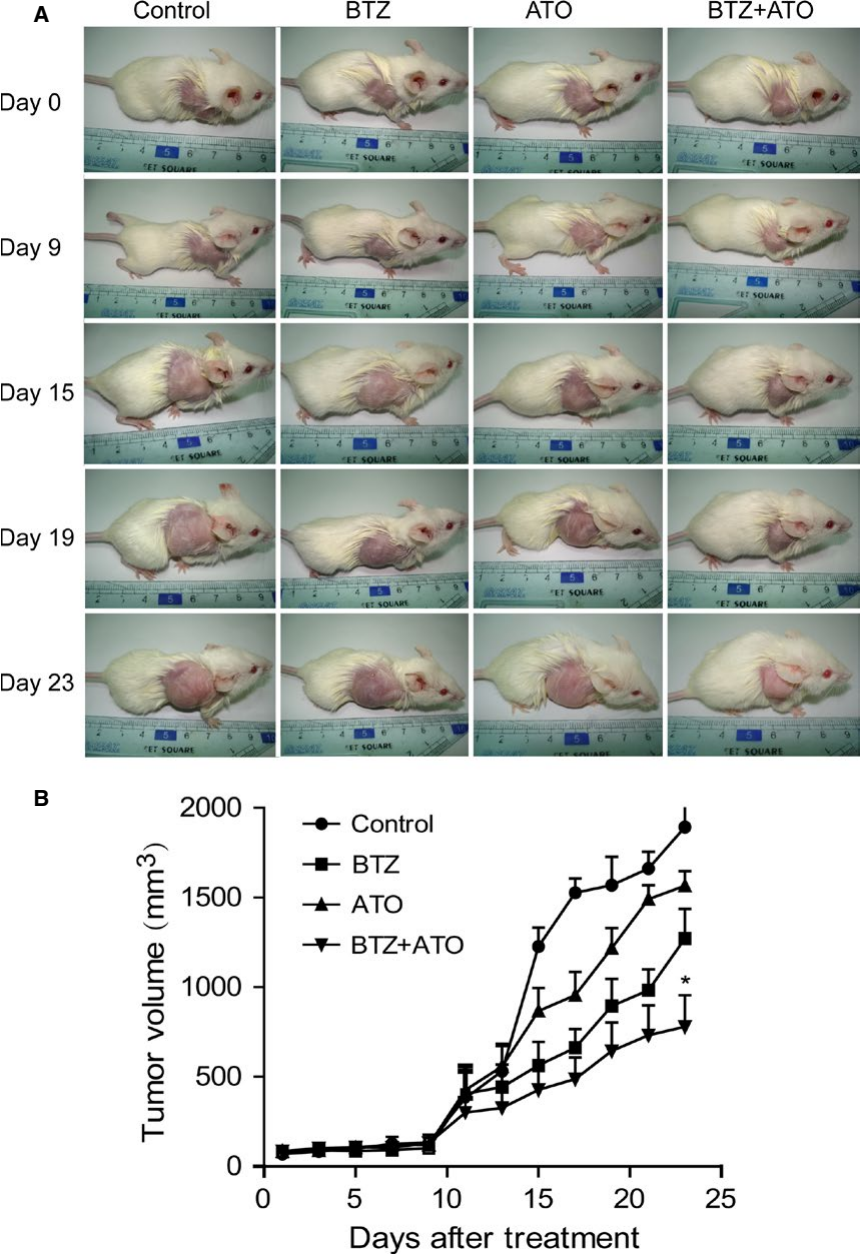
The in vivo efficacy of ATO combined with BTZ on MCL cells. (A) The pictures of mice taken at day 0, 9, 15, 19, and 23 after treatment. One representative picture from each group (control, BTZ, ATO, BTZ+ATO) was shown. (B) The volumes of tumor bulk of mice in each group altered over time. *P <* *0.05. ATO, arsenic trioxide; BTZ, bortezomib; MCL, mantle cell lymphoma.

To further investigate the effects of the ATO–BTZ combination, we established a tumor model by subcutaneous inoculation of Jeko-1 cells in NOD-SCID mice, and then carried out in vivo experiments. MCL-bearing mice were treated with NS control, BTZ alone, ATO alone, or the ATO–BTZ combination. The tumor volumes of mice in each group grew over time. We presented the sizes of tumors carried by these four groups of mice on day 0, 9, 15, 19, and 23 after treatment (Fig.6A). No remarkable difference was found in the first week when the tumor growth was rather slow. However, 2 weeks later, it was clearly demonstrated that the tumor volumes began to vary, and significant differences were found after 3 weeks (Fig.6B). Among them, the tumor volumes of the control group were the biggest, followed by that of the ATO group, then the BTZ group, while the minimal size was found in ATO–BTZ combination group. In the first 15 days, significantly different could be seen when each treatment group was compared to the control group, while no obvious difference among the treatment groups. However, on the 17th day, significant difference was found when the BTZ group and the combination group were compared to the control group and ATO group, although the differences were not significant at that time. With the increase in the days of treatment, the differences between BTZ group and the combination group became more and more obvious, and eventually to the 23rd day, a statistically significant difference could finally be documented between these two groups (the asterisk indicates a statistically significant difference, *P* < 0.05) (Fig.6A and B).

Throughout the experiment, the body weights of mice were decreased with time, but no significant difference was found among groups (Fig. S4). These data clearly demonstrated the in vivo anti-MCL activity of the ATO–BTZ combination.

## Discussion

Although most of the MCL patients respond well to BTZ treatment, relapse within a few years after remission is still a common outcome; meanwhile, the toxicities of BTZ in the course of treatment, especially peripheral neuropathy, forced us to reduce the drug dosage, which reduced the therapeutic effects as well. It is ideal to maintain or even improve the efficacy of BTZ while reducing its side effects. That is why we proposed to explore a new combination therapy based on the addition of another reagent to compensate to the weakness of BTZ using alone.

BTZ can increase the protein level of proapoptotic protein Noxa in MCL cells, thereby inducing the apoptosis of MCL cells. However, as a proteasome inhibitor, it also induces the antiapoptotic protein Mcl-1. Therefore, the new participant has to be able to downregulate the Mcl-1 to overcome the weakness of BTZ and to achieve a complementary effect. A variety of BH3 analogs have been developed, such as GX15-070, ABT-263, ABT-737, etc., which can inhibit the expression of antiapoptotic Bcl-2 family proteins (such as Mcl-1, Bcl-2, Bcl-XL, etc.). However, the BH3 analog that can be conjugated with Mcl-1 with high affinity and high specificity has not been developed yet 35–38. We reported for the first time ATO, an effective drug for APL, can downregulate the Mcl-1 expression in MCL 26. Recently, the studies of Wang et al. in NB4 cells supported our findings. Indeed, they found that ATO could downregulate the Mcl-1 level by following two mechanisms: first, it can activate GSK-3*β* by inhibiting the ERK/AKT pathways thereby increasing the degradation of Mcl-1; second, it prevents the phosphorylation of Thr163 in Mcl-1 by direct inhibition of the ERK pathway, thus decreasing the stability of Mcl-1 and downregulating the level of Mcl-1 34. All these studies provided us the theoretical support of the combination therapy of ATO and BTZ in MCL.

We found that ATO could inhibit the proliferation of MCL cell lines Jeko-1 and Granta-519 cooperatively with BTZ; combination of two drugs could also cooperatively induce the apoptosis of MCL cell lines, and the proapoptotic effect of ATO combined with low-dose BTZ was apparently stronger than that of relative higher dose of BTZ. We then validated it in the primary cells of MCL patients. The fraction of tumor cells in BMMCs of patient 1, 2, and 3 was 38.3%, 65.4%, and 10.5%, respectively. We examined the apoptosis of tumor cells (CD45+CD19+CD5+*κ*+ or CD45+CD19+CD5+*λ*+) and normal cells (CD45+CD19−) after treatment of single drug or two-drug combination. We found that the proapoptotic effect of ATO combined with BTZ was specific to tumor cells of MCL patients with minimal toxicity to normal cells. Being consistent with the results in cell lines, ATO combined with BTZ could significantly reduce the dosage of BTZ thus decreasing its toxicities while ensuring the anti-MCL efficacy. In patient 4, whose tumor cells dominated the BMMCs (93.1%), the same result was obtained from direct treatment by these drugs. These experiments strongly support a therapeutic strategy based on the treatment of MCL with a combination of ATO and BTZ.

ATO and BTZ may exert their antitumor effects by cooperatively adjusting the balance between proapoptotic proteins and antiapoptotic proteins. It is worth noting that Bcl-2 was downregulated after treatment by the two drugs in the Jeko-1 cells, while the expression of Bcl-2 in the Granta-519 cells did not change. This indicated that Bcl-2 did not play a common role in the apoptotic mechanism of combination therapy. After ATO was added, as compared with BTZ, the two-drug combination group maintained the high level of Noxa while the protein level of Mcl-1 was somewhat lower. Moreover, the ratio of total protein amount of Noxa and Mcl-1 was shown to be the lowest in the control group, whereas that ratio becomes highest in the two-drug combination group. Thus, the antiapoptotic protein Mcl-1 and proapoptotic protein Noxa may play an important role in the apoptosis cooperatively induced by the two drugs.

It is reported that by the interaction of Noxa or that of other BH3-only proteins with Mcl-1 or with Bcl-xl, Bak can be released from Mcl-1 or from Bcl-xl to play a proapoptotic role 32,33. When we further analyzed the interaction of Mcl-1/Noxa, Mcl-1/Bak, and Bcl-xl/Bak, it was found that when using BTZ alone or combination of the two drugs, the Noxa binding to Mcl-1 increased significantly in both cases, however, in the two-drug combination group, the amount of Noxa binding to Mcl-1 in combination group was not more than that in BTZ alone group, and this may be due to the reduced amount of Mcl-1 protein in the combination group compared to the BTZ alone group, which could be seen both in Jeko-1 cells and in the primary cells of patient 4. Meanwhile, it was found that in Jeko-1 cells, the amount of Bak conjugated with Mcl-1 or Bcl-xl was the lowest in the combination group as compared to the other groups, indicating that Bak was released from Mcl-1 and from Bcl-xl to exert its proapoptotic effect.

In the subsequent in vivo study, the MCL cell line Jeko-1 was used to establish a NOD-SCID mice xenograft model. In order to maximize the efficacy while minimizing side effects of drugs, we chose the combination treatment of ATO and BTZ. In this scenario, the tumor growth could be delayed most effectively, as compared with the control group, the group treated by BTZ alone or ATO alone.

In vitro and in vivo studies both indicated that ATO could play the anti-MCL role cooperatively with BTZ. Meanwhile, it was found that when combined with ATO, the anti-MCL effect of low-dose BTZ could be at least the same as that of relative higher doses BTZ alone, so as to reduce the dosage while avoiding or significantly reducing the peripheral neuropathies caused by BTZ, thus to solve the problem that has long plagued the clinicians. ATO used to be a second-line treatment for relapsed or refractory APL; however, it has gradually become the first-line choice in newly diagnosed APL 39,40. During treatment, small doses of ATO did not cause significant toxicity, and the liver or cardiac toxicity manifested in some patients was generally reversible, so we have a reason to believe that, in the treatment of MCL, ATO and BTZ can collaborate in clinical use, and the corresponding clinical trials will be conducted. We look forward to the good effect of combined therapy of these two drugs, which might be regarded as a classic example in translational therapy.

## Supporting Information

**Figure S1.** BTZ or ATO monotherapy inhibited the proliferation of MCL cells. MCL cell lines, Jeko-1 and Granta-519, were incubated with the indicated concentrations of BTZ (2.5, 5, 7.5, 10, 15, and 20 nmol/L) (A) or ATO (0.25, 0.5, 1, 2, and 5 *μ*mol/L) (B). After 12, 24, or 48 h, cell viability was determined by CCK8.

**Figure S2.** The conformational alterations of Bak (A)/Bax (B) in Jeko-1 and Granta-519 cells were examined after 48-h treatment of ATO, BTZ, and combination of the two drugs.

**Figure S3.** The proapoptotic effect of ATO combined with BTZ on PBMCs of healthy population. The apoptotic status of PBMCs from healthy donors was detected after 18-h treatment of ATO (2 *μ*mol/L) alone, BTZ (7.5 nmol/L) alone, or combination of the two drugs.

**Figure S4.** The body weights of mice in each group varied over time.
