# Supplementary figures and images for: Arsenic trioxide rewires mantle cell lymphoma response to bortezomib
Source: Cancer Med. 2015 Aug 26;4(11):1754–66. doi: 10.1002/cam4.511 (PMC4674002; doi:10.1002/cam4.511)

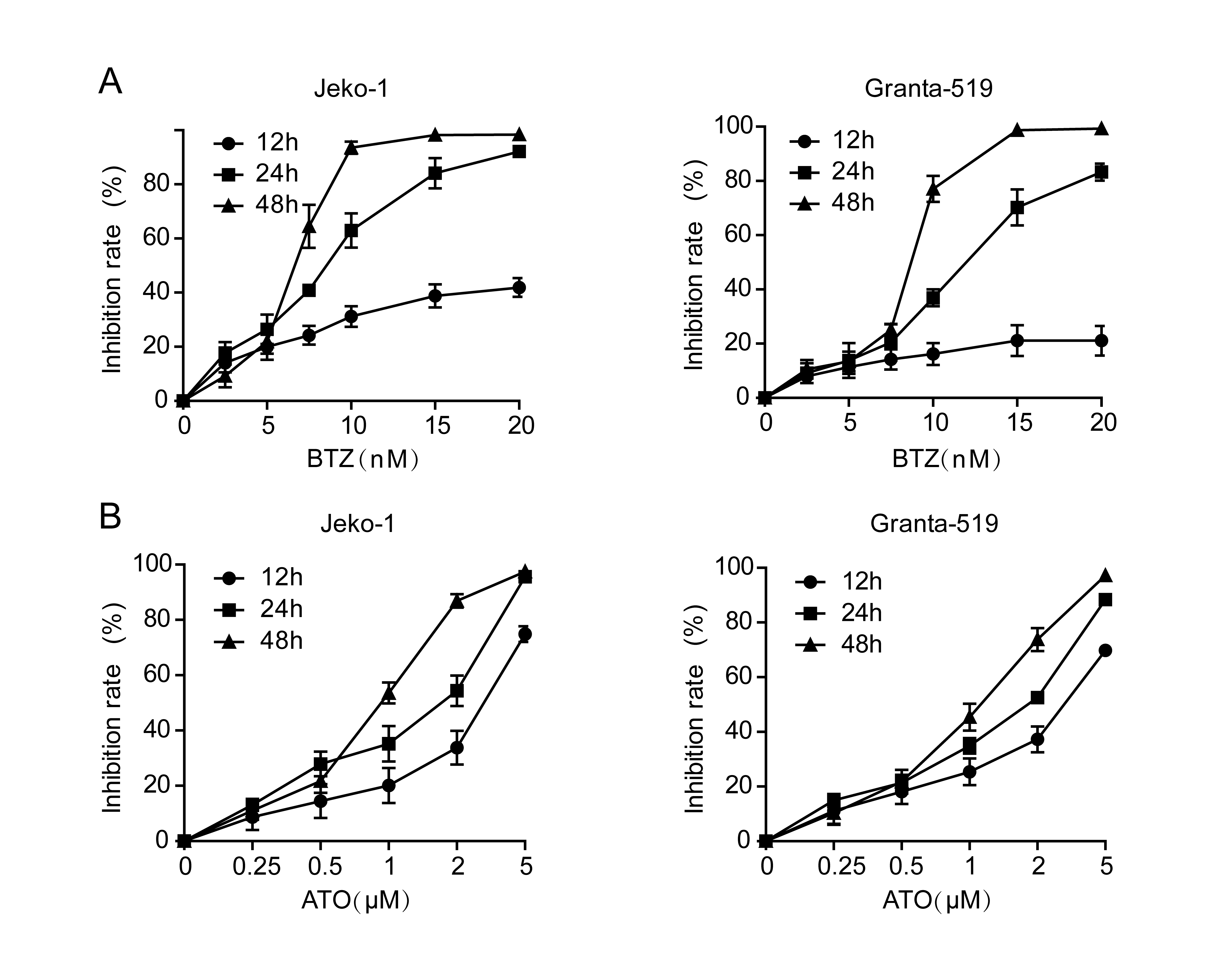

Supplement: Supplementary file 1 [file cam40004-1754-sd1.tif]

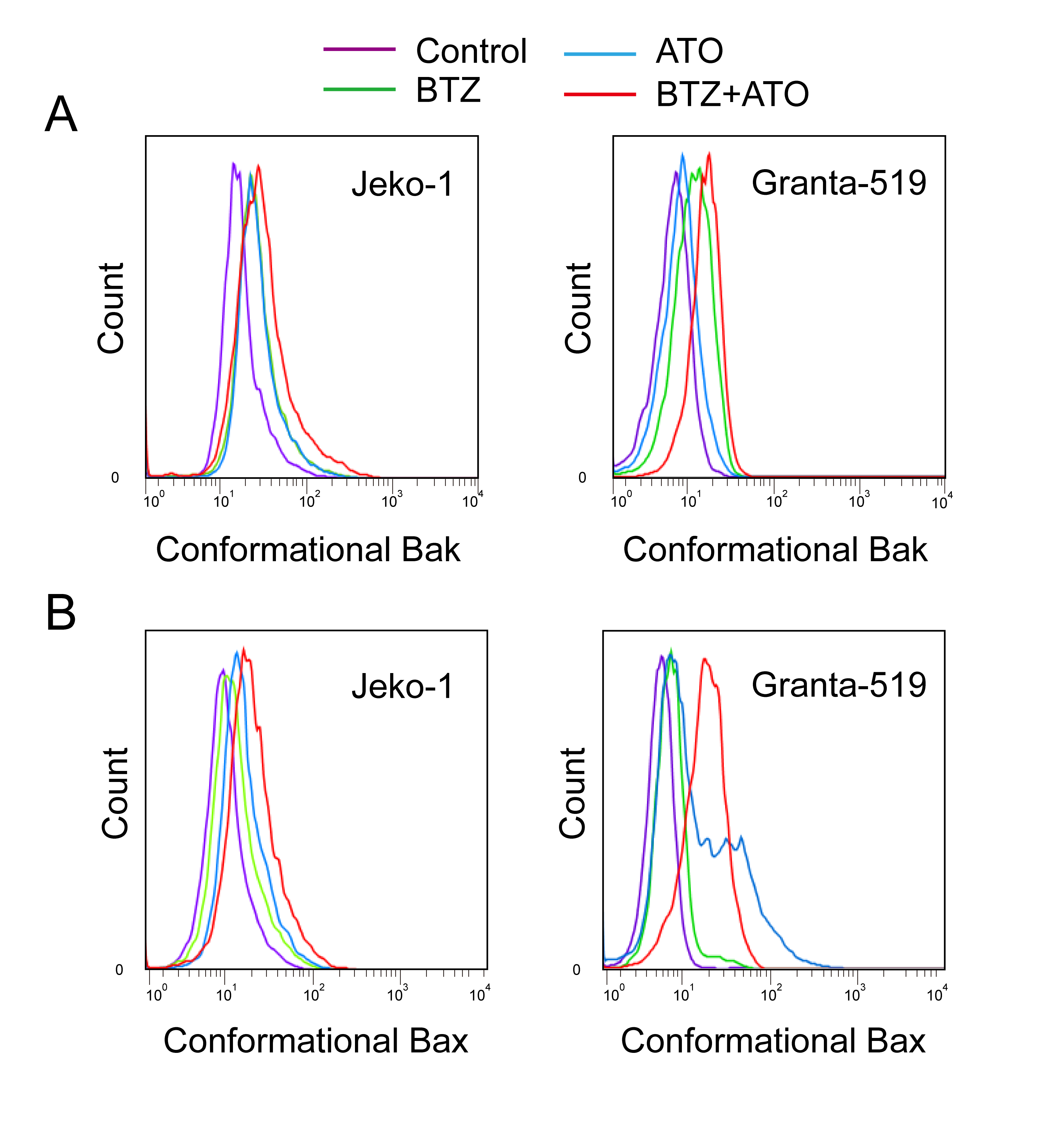

Supplement: Supplementary file 2 [file cam40004-1754-sd2.tif]

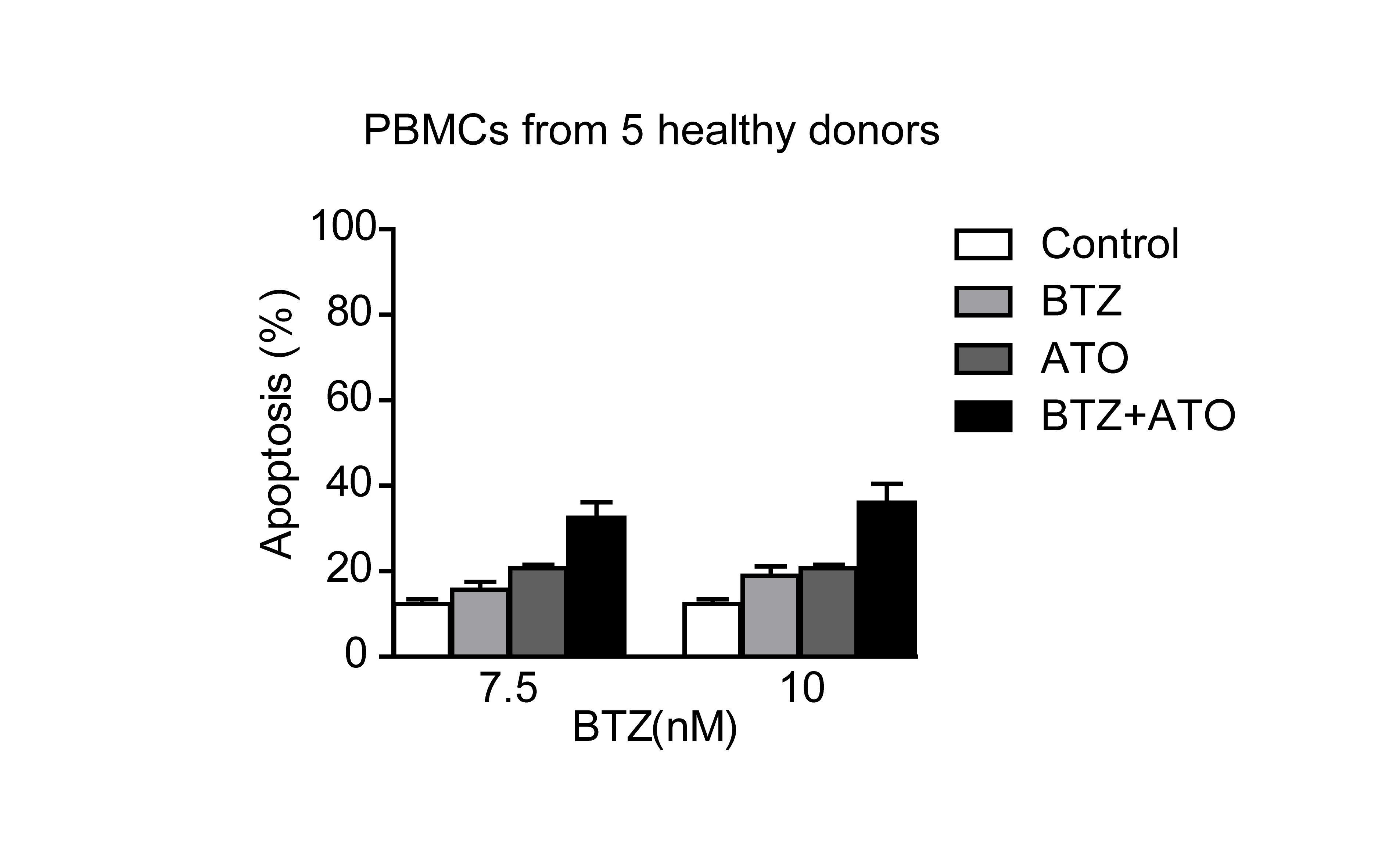

Supplement: Supplementary file 3 [file cam40004-1754-sd3.tif]

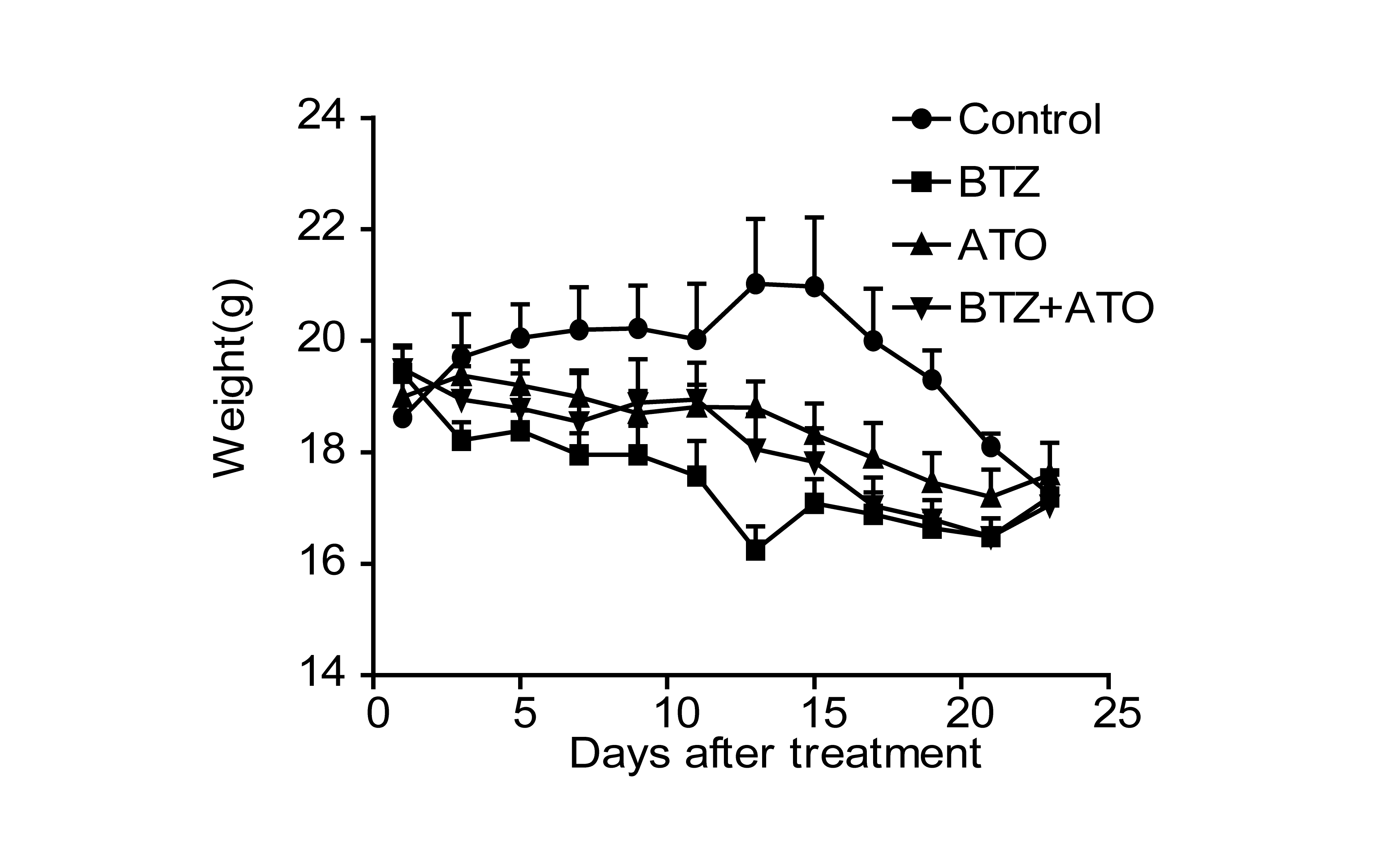

Supplement: Supplementary file 4 [file cam40004-1754-sd4.tif]
